# Serologic Investigations in Children with Inflammatory Bowel Disease and Food Allergy

**DOI:** 10.1155/2009/512695

**Published:** 2009-12-06

**Authors:** Urszula Grzybowska-Chlebowczyk, Halina Woś, Aleksander L. Sieroń, Sabina Więcek, Aleksandra Auguściak-Duma, Halina Koryciak-Komarska, Joanna Kasznia-Kocot

**Affiliations:** ^1^Gastroenterology Division, Department of Paediatrics, Medical University of Silesia, 40-752 Katowice, Poland; ^2^Department of General, Molecular Biology and Genetics, Medical University of Silesia, 40-752 Katowice, Poland

## Abstract

The aim of the study was the evaluation of frequency and titre of IgA ASCA and IgG ASCA and p-ANCA, c-ANCA in children with IBD and occurrence of ASCA antibodies in relation to coexistence of FA. Patients and methods. The study comprised 95 children at the ages of 2 to 18 years. The diagnosis of IBD was established on the basis of Porto criteria. Tests of blood serum were performed in all children: IgA and IgG ASCA, p-ANCA, c-ANCA using ELISA method. Results. IgE-dependent FA was found in 32.5% children with UC and in 21% with CD. We did not observe any relation between the occurrence of FA and the frequency and ASCA titre. p-ANCA were significantly more frequent in the group of children with UC. The occurrence of ASCA antibodies was observed in 73.7% of children with CD, 17.5% with UC and almost 30% with allergic colitis. Conclusions. Patients with CD and the presence of ASCA revealed a significantly more frequent localization of lesions within the small bowel and a tendency towards older age. We observed a connection between the occurrence of antibodies and the examined mutations of gene NOD2/CARD15.

## 1. Background

In the recent years we have observed not only an increased incidence of Inflammatory Bowel Disease, especially Crohn's disease in children, but also other, less frequent in this age group, types of colitis, including allergic colitis. Allergic colitis is a rare type of inflammation occurring in the course of IgE-mediated or IgE-independent food allergy, more frequently it affects children. This disease occurs in two age groups: in infancy or during puberty and in young adults. In the case of allergic colitis of adult type, clinical symptoms depend on the degree of intensity and the localization of inflammation—the predominant symptoms are those that are typical for Inflammatory Bowel Disease—chronic diarrhea, abdominal pain, loss of weight, and lack of appetite [1, 2]. In endoscopy the mucosa may be free of any lesions or may show non-characteristic inflammatory changes, such as reddening, unclear vascular markings, or hypertrophy of lymph nodules; the changes are often focal with eosinophilic infiltration within the mucosa and sometimes with the presence of multinuclear giant cells in the submucosa. In the case of a big intensity of eosinophilic infiltration we call the disease eosinophilic colitis [3]. 

Eosinophils occur not only in the course of allergic colitis, but also in the course of other inflammatory processes involving the bowels as well as in drug-induced reactions [4]. Both IBD and food allergy are the results of interactions between immunologic, genetic and environmental factors. Coexistence of food allergy is also frequently mentioned in the course of ulcerative colitis and Crohn's disease; many studies also indicate a connection between allergic colitis and later development of IBD [5, 6].

ASCA antibodies against Saccharomyces cerevisiae and ANCA antibodies against the cytoplasm of neutrophils have been described since 1989, as being present in the serum of patients with Inflammatory Bowel Disease, and now they are considered to be a subclinical, genetic marker of a high risk of development of Crohn's disease and ulcerative colitis, respectively. The frequency of ASCA antibodies is estimated as 40%–70% in Crohn's disease and 5%–20% in ulcerative colitis, and the frequency of ANCA antibodies is estimated as 40%–80% in ulcerative colitis and 15%–20% in patients with Crohn's disease, especially with involvement of the large bowel (UC-like) [7–9].

There is a connection between a clinical phenotype of CD and ASCA—patients with a high titre of ASCA antibodies in blood serum more frequently manifest a grave course of disease with fibrostenosis and perforations, which may be associated with localization within the small bowel, whereas no link between p-ANCA antibodies and the clinical picture of ulcerative colitis was found [10].

On the basis of the current diagnostic methods, almost 10% of patients with Inflammatory Bowel Disease are initially wrongly classified. In the next 10%, the final diagnosis is difficult to be established, and indetermined colitis is diagnosed [11]. It seems that the determination of ASCA and p-ANCA in various inflammatory bowel diseases will allow to specify and establish the diagnosis.

*The aim of the study* was

1. The evaluation of frequency and titre of antibodies against Saccharomyces cerevisiae (IgA ASCA and IgG ASCA) and anti-neutrophilic (p-ANCA and c-ANCA) in children with various forms of Inflammatory Bowel Disease.
2. The evaluation of ASCA antibodies occurrence in relation to coexistence of food allergy in children with ulcerative colitis and Crohn's disease.

## 2. Patients and Methods

The study comprised 95 children at the ages of 2 to 18 years (mean age 12.6), *78 with Inflammatory Bowel Disease and 17 with allergic colitis*. The diagnosis of IBD was established on the basis of Porto criteria (physical examination, laboratory tests, immunological, radiological examinations, endoscopy of the upper and lower part of the alimentary tract and histopathological examinations) [12].

The examinations were carried out in the following groups:

 Group I-38 children with Crohn's disease
 Group II-40 children with ulcerative colitis
 Group III-17 children with allergic colitis
 Group IV-comparative-23 children with functional disorders of the alimentary tract, the most frequently caused by lactose intolerance, in whom performed examinations excluded the inflammatory background of reported complaints.

*Tests of blood serum* were performed in all children, in both the examined and control groups:**

(i) IgA and IgG ASCA—immunoenzymatic method (ELISA), using reagents produced by Euroimmune (norm up to 20 RU/mL)
(ii) p-ANCA, c-ANCA—immunoenzymatic method, using reagents produced by Biomedica (norm up to 3 U/mL)
(iii) IgE total and IgE specific for selected foods (milk, wheat flour, chicken, egg white and yolk)—immunofluorescent method (determination in immunological reaction of chemiluminescence), using reagents produced by DPC Kraków (norm for IgE total <87 IU/mL, norm for food-specific IgE <0.35 KU/I).

The mutations R702W, G908R and L1007fs of gene NOD2/CARD15 were determined in all examined children. [13].

### 2.1. Ethical Issues

In order to conduct this study the approval of the Bioethical Committee at the Medical University of Silesia in Katowice was obtained. All parents received the information about the study and they gave their written consent to participate. The informed consent was also obtained from those children who were over 16 years of age.

## 3. Results

### 3.1. Correlation between Clinical Phenotypes and Laboratory Tests

IgE-dependent food allergy was found in 13 children (32.5%) with ulcerative colitis and in 8 (21%) children with Crohn's disease. Although we did not find statistically significant differences in the occurrence of food allergy in children with ulcerative colitis and Crohn's disease, it is noticeable that atopy (IgE total) occurs more often in children with Crohn's disease, and allergy to particular foods is more frequent among children with ulcerative colitis. *In the control group, the features of atopy in the form of increased total IgE levels were found in 3 children (13%); none of these children had specific IgE*.

We did not find the influence of food allergy on the clinical picture of ulcerative colitis; however, we observed a tendency towards a more frequent occurrence of inflammatory changes in the duodenum with inflammatory infiltration consisting of lymphocytes, plasmatic cells and eosinophils in patients with Crohn's disease and food allergy. We did not observe any relation between the occurrence of food allergy and the frequency and ASCA titre (Table 1).

### 3.2. Immunologic Examination

We analyzed the presence of antibodies p-ANCA (+)/ASCA(−) and p-ANCA (−) and ASCA (+) in the group of children with allergic colitis and we determined the presence of these antibodies in 47% of patients. We found significant differences in the frequency and titre of ANCA and ASCA between particular groups (Tables 2 and 3).

Statistically, antibodies against p-ANCA were significantly more frequent in the group of children with ulcerative colitis in comparison with the control group. These antibodies were found in 25% of children with ulcerative colitis, 10% of children with Crohn's disease and 17.5% of children with allergic colitis. Sensitivity of p-ANCA for diagnosing UC equals 25%, specificity (in relation to controls) was 100%.

We did not observe any relation between the occurrence of p-ANCA and the clinical picture of ulcerative colitis and allergic colitis; in Crohn's disease these antibodies were found only in children with disease localization in large bowel.

The occurrence of ASCA antibodies (IgA and/or IgG) was observed in 73.7% of children with Crohn's disease, 17.5% with ulcerative colitis and almost 30% with allergic colitis, statistically significantly more frequent in children with Crohn's disease in comparison with the other examined groups and controls. Statistically, patients with Crohn's disease and the presence of ASCA revealed a significantly more frequent localization of lesions within the small bowel and a tendency towards older age (mean age 15.5). Sensitivity ASCA (IgG or IgA) for diagnosing Crohn's disease was determined as 73.7%, specificity (in relation to controls) was 91.3%.

Sensitivity of IgA ASCA for diagnosing Crohn's disease was determined as 57.9%, specificity (in relation to controls) was 95.7%.

Sensitivity of IgG ASCA for diagnosing Crohn's disease was determined as 71.1%, specificity (in relation to controls) was 95.7%.

### 3.3. Molecular Examination

In the group of children with Crohn's disease, 15 patients (39.5%) were carriers of at least one mutation of CARD15 gene; among them the most frequent was R702W mutation in 7 children (18.4%), L1007fs mutation in 6 children (15.8%), the least frequent was G908R mutation in 3 children (7.9%). We presented the occurrence of ANCA and ASCA in relation to mutations R702W, G908R, and L1007fs in the examined groups of children. We observed a connection between the occurrence of antibodies and the examined mutations of gene NOD2/CARD15 (Table 4).

## 4. Discussion

In the course of inflammatory bowel diseases like Crohn's disease, allergic and ulcerative colitis, the inflammatory process results from disorders of the immune system regulation. The mucosa becomes damaged due to a pathological response of T lymphocytes, secreted cytokines and cytotoxic activity of effector cells. Th1 response with increased secretion of TNFalpha, IFNgamma and other proinflammatory cytokines is characteristic for the active phase of Crohn's disease, celiac disease and IgE-independent allergy (i.e., cellular type). Whereas, in ulcerative colitis and IgE-mediated allergy a predominant role is played by a subpopulation of Th2, producing cytokines responsible for humoral type of response with increased synthesis of antibodies, especially IgE and IgG. In allergic colitis a more predominant type of response is cellular one; however, the pathomechanism is not completely established yet [14–16].

Eosinophilic infiltration in the large bowel is a common change in many diseases, like classic IgE mediated food allergy, allergic colitis or Inflammatory Bowel Disease. In IBD eosinophils usually comprise a small percentage of predominant lymphocytic infiltration, but their high level is a predictor of unfavourable prognosis, since the products of their degranulation, such as eosinophil cationic protein, have a proinflammatory effect and damage tissues; degranulation of eosinophils was also observed in the course of IBD [17, 18]. At present, IgE-mediated and IgE-independent food allergies are taken into account in the pathogenesis of allergic colitis. Eosinophilic infiltration of III degree according to Whitington was found in our examined group in 4 children with Crohn's disease, in 2 with ulcerative colitis and in 6 with allergic colitis. It is known that food allergy occurs in the course of *Inflammatory Bowel Diseases* especially in children. IgE-mediated food allergy is more frequently described in colitis ulcerative, probably in relation to the described above pathomechanism of this disease, however, it is also observed in patients with Crohn's disease [19–21]. Perhaps in this group of patients IgE-independent allergy occurs more frequently (like in allergic colitis). Among our examined patients IgE-mediated allergy specific to particular food was diagnosed in 21% of patients with Crohn's disease and in over 32% of patients with ulcerative colitis; the features of atopy in the form of sole high IgE was found twice more often in patients with Crohn's disease, they are probably connected with immunologic disorders occurring in the course of disease; we obtained similar results in the previously published studies [22]. Considerably lower frequency of food allergy was observed by Bartukowa—in 14.3% patients with Crohn's disease and in 8.7% of patients with ulcerative colitis [23]. We determined elevated values of ASCA in 30% of patients with allergic colitis, in 70% with Crohn's disease and in 10% with ulcerative colitis. Statistically, the values of ASCA were significantly higher in Crohn's disease. In children with Crohn's disease and food allergy we observed a tendency towards a more frequent duodenitis. In 2 children with previously diagnosed eosinophilic colitis and high titre of ASCA, after 6 and 12 months of disease duration we diagnosed Crohn's disease. Among children with allergic colitis and the presence of ASCA antibodies, one child has a positive family history towards Crohn's disease, and in one child a severe course of disease suggests a future development of Crohn's disease, in both patients we did not find any mutations of gene NOD2/CARD15. Anti Saccharomyces cerevisiae antibodies are regarded as a serologic marker of Crohn's disease; they occur in 40–70% of patients, mainly in the form of disease with the involvement of the small bowel and the early onset of the disease (investigations were performed in adult patients) [24, 25]. Similarly, in our studies we observed a relation between ASCA antibodies occurrence and the localization of disease in the small bowel; however, the mean age of children with ASCA was higher (15.5 years) in comparison with the group without these antibodies (11.5 years). Israeli et al. observed growing values of ASCA in asymptomatic subjects in the period before establishing the diagnosis of CD; ASCA were present in 31% of patients before the diagnosis; the most often they appeared 36 months before the manifestation of clinical symptoms. Israeli suggests regarding the presence of ASCA to be an early marker of the disease or a marker of immunologic response to environmental antigens in the context of the early stage of disease. The presence of ASCA in the high risk group may be a marker of CD development and may even predict its clinical course. Even asymptomatic patients with the presence of ASCA should be monitored for CD [26]. ASCA are also found in the course of different diseases, like celiac disease. So far the tests for the presence of anti ASCA antibodies in patients with food allergy have not been made. The results of production of ASCA are unknown. It is assumed that increased permeability of the small bowel in patients with Crohn's disease may lead to increased exposure of yeast antigens (which are a basic component of normal intestinal flora) to immunologic response cells. This hypothesis could also explain the presence of ASCA in the course of celiac disease and food allergy, which are inseparably connected with increased permeability of the small bowel mucosa. Basta observed the occurrence of ASCA in 30% of patients with celiac disease, whereas Granito found ASCA in 59% of patients with celiac disease, more often in class IgG, and their disappearance after introducing a gluten-free diet [27, 28]. He also discovered the presence of ASCA in several people with latent celiac disease, without clinical symptoms (healthy relatives), like Israeli did in the case of Crohn's disease [14]. Granito formed a hypothesis that ASCA are a result of nonspecific immunologic response to a prolonged damage of the small bowel, and not a specific marker of Crohn's disease. In our study, titres of ASCA in children with food allergy were significantly lower than in Crohn's disease and they mainly occurred in class IgG. We did not observe a more frequent occurrence of ASCA and ANCA antibodies in children with food allergy in the course of Crohn's disease and ulcerative colitis nor a connection between food allergy and ASCA and p-ANCA antibodies. Polymorphism of gene NOD2/CARD15 is connected with the same phenotype of the disease as ASCA; however, Halfarson did not find any connection between the three most important mutations of gene CARD15 and the presence of ASCA [29, 30]. In our examination we found a relation between the examined mutations of gene NOD2/CARD15 and the presence of antibodies. Similary Annese in a large group of patients with Crohn's disease proved a relation between mutation NOD2/CARD15 and the localization in the small bowel, the narrowing form of the disease and the early age of diagnosis, but she also showed a relation between the mutation and the presence of ASCA [31]. The presence of ASCA is important for patients with undetermined colitis, however, 50% of these patients do not produce ASCA and p-ANCA.

The presence of ASCA+/ANCA− allows to predict Crohn's disease in 80% of patients with undetermined colitis, and the pattern ASCA−/ANCA+ predicts ulcerative colitis in 64% of these patients [32–35].

 The examined children in whom we diagnosed allergic colitis, performing serologic examinations of ASCA and ANCA, would allow to establish precisely the diagnosis in almost half of children (47%). In several patients both the clinical picture and the results of the other examinations could suggest IBD from the very beginning, whereas the majority of the examined children were qualified to the group with allergic colitis with regard to normal parameters of inflammation and the absence of changes in colonoscopy.

In the case of our children with allergic colitis it is not known whether elevated ASCA titres are connected with the small bowel damage, as Granito observed in celiac disease, and they disappear after normalization of changes within the small bowel, or maybe they are an early serologic symptom of Crohn's disease, as was observed by Israeli in the case of diagnosed colitis. It seems that only a long-lasting clinical and serologic observation of these patients will help to establish the proper diagnosis.

## 5. Conclusions

We proved a high specificity of ASCA antibodies for Crohn's disease and p-ANCA for ulcerative colitis. We observed a relation between the occurrence of ASCA antibodies and localization in the small bowel, and a relation between p-ANCA and localization in the large bowel in children with Crohn's disease, and also a connection between food allergy and duodenitis in children with Crohn's disease.

We did not prove any relation between ASCA antibodies and the occurrence of food allergy in the examined groups of children. In the course of allergic colitis, p-ANCA and ASCA antibodies are present in almost half of children, which may direct diagnosis towards ulcerative colitis or Crohn's disease. The performance of genetic and serologic examinations in the group of children with colitis related to food allergy allows to separate a group of patients requiring a strict gastrological observation and control examinations in order to establish the final diagnosis of CD or UC.

## Figures and Tables

**Table 1:** Relation between the frequency of occurrence and titre of ASCA antibodies and food allergy.

	Allergy (+)	Allergy (−)	*P*
ASCA (IgG or IgA) in whole examined group	40.8%	43.5%	.84
ASCA (IgG or IgA) in UC	17.7%	17.4%	1.0
ASCA (IgG or IgA) In CD	80%	69.6%	.71
Titre (median, range) IgA ASCA in whole examined group	7.7 (0–200)	9.1 (0–200)	.69
IgG ASCA in whole group	10 (0–200)	10.1 (0–200)	.96
IgA ASCA in UC	6.8 (0–200)	6.8 (0–200)	.85
IgG ASCA in UC	5.4 (0–200)	4.6 (0–200)	.86
IgA ASCA in CD	114.5 (2.9–200)	61.4 (0–200)	.59
IgG ASCA in CD	87.3 (0–200)	68.2 (0–200)	.41

**Table 2:** Comparison between the frequency of occurrence of ANCA and ASCA antibodies in groups.

	Controls	UC	CD	Allergy colitis	*P*
pANCA (+) (%)	—	25	10.5	17.6	.04*
cANCA (+) (%)	—	10	—	—	.04**
IgA ASCA (+) (%)	4.3	7.5	57.9	17.6	<.00001***
IgG ASCA (+) (%)	4.3	17.5	71.1	29.4	<.00001****
ASCA IgG and/or IgA (%)	8.7	17.5	73.7	29.4	<.00001*****
Combinations ANCA and ASCA (%)					<.00001******
pANCA(+)ASCA(+)	0	2.5	5.3	0	
pANCA(+)ASCA(−)	0	22.5	5.3	17.7	
pANCA(−)ASCA(+)	8.7	15	68.4	29.4	
pANCA(−)ASCA(−)	91.3	60	21.1	52.9	

*UC versus Ctrl, *P* = .01.

**In exact Fisher's test there are not any significant relations while comparing groups in pairs.

***CD versus Ctrl, *P* < .00001; CD versus UC, *P* < .00001; CD versus AC, *P* = .008.

****CD versus Ctrl, *P* < .00001; CD versus UC, *P* < .00001; CD versus AC, *P* = .007.

*****CD versus Ctrl, *P* < .00001; CD versus UC, *P* < .00001; CD versus AC, *P* = .003.

******Concerning combination pANCA(−)ASCA(+): CD versus Ctrl, *P* < .00001; CD versus UC, *P* < .00001; CD versus AC, *P* = .009.

**Table 3:** Comparison between titres of ANCA and ASCA antibodies in groups.

	Controls	UC	CD	Allergy colitis	*P*
Titre (median, range) pANCA (+)	0 (0–3.8)	0 (0–27)	0 (0–14.4)	0 (0–6)	.048*
cANCA (+)	0 (0–0)	0 (0–10.4)	0 (0–3.3)	0 (0–4.2)	.006**
IgA ASCA (+)	5 (0–28.2)	6.8 (0–200)	62.7 (0–200)	7.1 (2.2–125)	<.0001***
IgG ASCA (+)	4.6 (0–36.3)	4.9 (0–200)	79.3 (0–200)	9 (0–90.6)	<.0001****

*UC versus Ctrl, *P* = .03;

**UC versus Ctrl, *P* = .03;

***CD versus Ctrl, *P* = .000001; CD versus UC, *P* = .000001; CD versus AC, *P* = .0008.

****CD versus Ctrl, *P* = .00003; AC versus Ctrl, *P* = .02; CD versus UC, *P* = .00008; CD versus AC, *P* = .007.

**Table 4:** The presence and titre of p-ANCA and ASCA in relation to the occurrence of R702W/G908/L1007fs mutation in Crohn's disease.

	Positive mutation *N* = 15	Lack of mutation *N* = 23	*P*
pANCA (+) (%)	6.7	13	1.0
IgA ASCA (+) (%)	73.3	47.8	.18
IgG ASCA (+) (%)	86.7	60.9	.14
ASCA IgG i/lub IgA (%)	93.3	60.9	.06
pANCA (+) – (median, range)	0 (0–11.7)	0 (0–14.4)	.36
IgA ASCA (+) – (median, range)	200 (6.4–200)	18.7 (0–200)	.09
IgG ASCA (+) – (median, range)	150.9 (7.1–200)	34.9 (0–200)	.01
